# Congenital Hemihyperplasia in an Infant with Ipsilateral Torticollis: A Case Report

**DOI:** 10.3390/children10081352

**Published:** 2023-08-06

**Authors:** Jun Woo Kim, Yu Chan Park, Seung Hoon Han

**Affiliations:** 1 Department of Rehabilitation Medicine, Hanyang University College of Medicine Medical Center, 222-1, Seoul 04763, Republic of Korea; vigroth@naver.com (J.W.K.); yuchan6769@naver.com (Y.C.P.); 2 Department of Rehabilitation Medicine, Hanyang University Guri Hospital, 249-1, Guri-si 11923, Republic of Korea

**Keywords:** hemihyperplasia, torticollis, sternocleidomastoid muscle, embryonal tumor, ultrasound, physical therapy

## Abstract

Hemihyperplasia is a kind of regional body growth asymmetry and can be a symptom of several congenital disorders and tumorous conditions. Torticollis is most commonly caused by asymmetric hypertrophy of the sternocleidomastoid muscle. Herein, we report a case of hemihyperplasia in an infant with ipsilateral torticollis. The baby was evaluated using physical examination and ultrasonography. We observed significant right-side torticollis that was ipsilateral to congenital right-side hemihypertrophy. No abnormal tumorous conditions were found during the evaluation in the pediatrics department. The patient was treated with physical therapy and exhibited mild improvements in torticollis and hemihyperplasia.

## 1. Introduction 

Congenital hemihyperplasia is defined as a kind of regional body growth asymmetry due to differences in the growth of bone, soft tissue, or both. It is also known as hemihypertrophy or overgrowth syndrome. Hemihyperplasia itself can be a symptom of several congenital syndromes, such as Beckwith–Wiedemann; Russell–Silver syndrome; Proteus syndrome; hemihyperplasia–multiple lipomatosis syndrome; or Klippel–Trenaunay–Weber syndrome; neurofibromatosis; or of tumorous conditions [1]. Isolated hemihyperplasia can be diagnosed in the absence of other symptoms [2]. It is differentiated from hemiatrophy where there is an undergrowth of usually just one body area; in contrast, hemihyperplasia affects multiple limbs and organs simultaneously [3].

Diagnosis of congenital isolated hemihyperplasia is difficult due to the lack of clinical criteria. To diagnose hemihyperplasia, there should be apparent regional body asymmetry. Arbitrarily, the growth discrepancy has to be greater than 5% [4]. This may include differences in the circumference or length. Since hemihyperplasia is related to several other syndromes, diagnosing congenital isolated hemihypertrophy involves the elimination of these other syndromes. Clinicians need to carefully assess and eliminate the possibility of other underlying conditions before confirming a diagnosis of congenital isolated hemihypertrophy [2]; however, a diagnosis is very important because hemihyperplasia is associated with risk of embryonal tumors like Wilms tumor and hepatoblastoma [1]. Therefore, all children with hemihyperplasia should undergo screening to detect tumorous conditions. Also, abdominal ultrasonography and serum alpha fetoprotein measurements should be performed every 3 months [2,5].

Congenital muscular torticollis is a relatively common musculoskeletal condition seen in infants, with common causes of asymmetric hypertrophy and contracture or aplasia of the sternocleidomastoid (SCM) muscle [6,7]. It can lead to an ipsilateral tilt and contralateral rotation of the face and chin. Other known causes include non-muscular torticollis, congenital fourth nerve palsy with ocular torticollis, Sandifer’s syndrome, neural axis abnormalities, acquired muscle damage, infections, and tumors [6]. It is important to identify the causes of torticollis, as some can be life-threatening. Congenital muscular torticollis is a relatively benign condition compared to non-muscular torticollis; however, delayed diagnosis and treatment can lead to developmental delays. Proper physical examinations and radiographic evaluations are required for early diagnosis [6]. To our knowledge, this is the first report of muscular torticollis with hemihyperplasia, here presenting on the ipsilateral side.

## 2. Case Description

**Figure 1:**
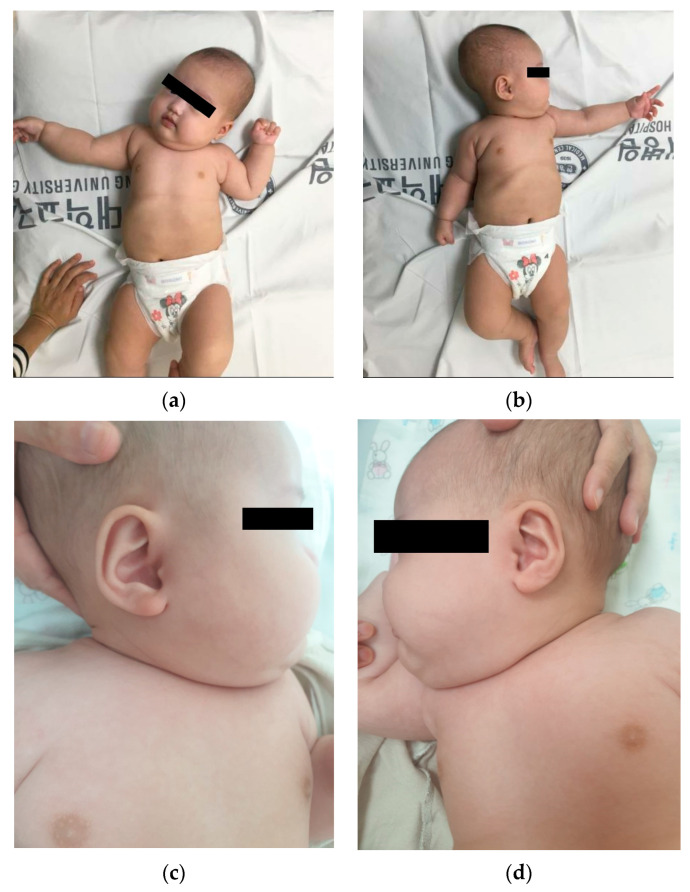
Gross photo of the baby exhibiting significant right-side hemihyperplasia: (**a**) Asymmetric tonic neck reflex of the right side with right-side hemihyperplasia; (**b**) Asymmetric tonic neck reflex of the left side; (**c**) Gross examination of the right side of the head and neck with decreased range of movement of the neck toward the left side; (**d**) Gross examination of the left side of the head and neck.

A 2-month-old female infant and her parents visited the developmental rehabilitation outpatient clinic for torticollis. The baby was the couple’s first child, born at a gestational age of 37 weeks with a birth weight of 2.9 kg. She was delivered via normal spontaneous vaginal delivery and exhibited no perinatal issues. There was no family history of genetic diseases or tumorous conditions. Torticollis was present at birth and became more prominent as the baby grew. Along with torticollis, the right side of her body exhibited more severe hyperplasic changes compared to the left side. A physical examination confirmed spontaneous movement, and the muscle tone of all four limbs was normoactive. The baby demonstrated head control only on the right side of her body but fine movement was normal for her age. In addition, the baby could babble and respond to surrounding noise. The body weight of the baby was 6.5 kg, which was proportional to her developmental age. No abnormal skin lesions, such as pityriasis rubra pilaris, café au lait spots, lisch nodules, axillary freckling, and cerebriform connective tissue nevus, were observed. Her deep tendon reflexes were normal, and no pathologic reflexes were seen. Asymmetric tonic neck reflex was observed bilaterally. No definite developmental delay was present, and there were no signs of facial dysmorphism, large tongue, omphalocele, ear creases, or other abnormalities. Additionally, no abnormal masses, lipomas, or vascular malformations were detected. The baby had grossly significant hemihyperplasia of the right upper and lower limbs (Figure 1). The size of the left upper and lower limbs appeared to be proportional to her developmental age. The baby’s fingers and toes were intact, and no macrodactyly or syndactyly was found. We measured the mid-arm and mid-thigh circumferences of both upper and lower limbs, which revealed that the right upper arm and thigh circumferences were 12% larger than those of the left limbs. The mid-arm circumference on the right side was 153 mm, and on the left side, it was 136 mm. The mid-thigh circumference on the right side was 245 mm, and on the left side, it was 215 mm. Asymmetric growth of the right upper and lower limbs was only found during gross examination.

**Figure 2:**
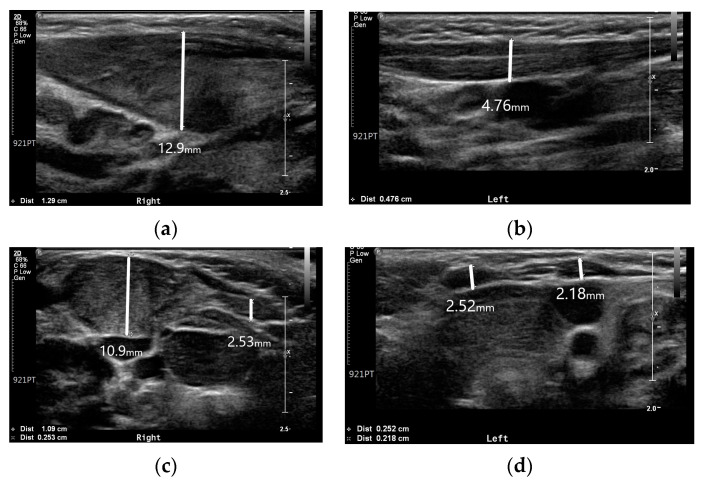
Neck ultrasonography of the SCM muscle: (**a**) Thickness of the right SCM muscle at mid-level; (**b**) Thickness of the left SCM muscle at mid-level; (**c**) Thickness of both SCM muscles at the clavicular insertion site; (**d**) Thickness of both SCM muscles at the sternal insertion site.

Ultrasound is the imaging modality of choice for radiographic evaluation of congenital muscular torticollis [8]. On ultrasound images, the SCM muscle appears as a relatively hypoechoic mass with echogenic lines; however, in cases of congenital muscular torticollis, the thickened SCM muscle mass can affect the size and signal intensity of the muscle. In conditions of congenital muscular torticollis, the affected SCM muscle looks thicker than the normal contralateral side, and the signal intensity of the muscle tends to be more hyperechogenic [8]. Neck ultrasonography exhibited asymmetric thickening of the right SCM muscle with hyperechogenicity (Figure 2). The thickness values of the SCM muscle at the right and left mid-levels were 12.9 and 4.7 mm, respectively, while the thickness values at the clavicular insertion point on the right and left sides were 10.9 and 2.1 mm. At the sternal insertion point, the right- and left-side thickness values were both 2.5 mm, and the baby exhibited prominent right-side muscular torticollis with fibromatosis coli.

**Figure 3:**
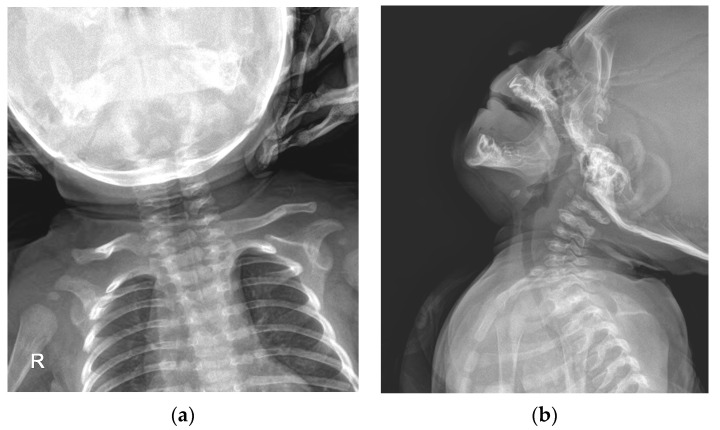
Cervical spine simple radiograph: (**a**) Anteroposterior view of the cervical spine; (**b**) Lateral-extension view of the cervical spine.

**Figure 4:**
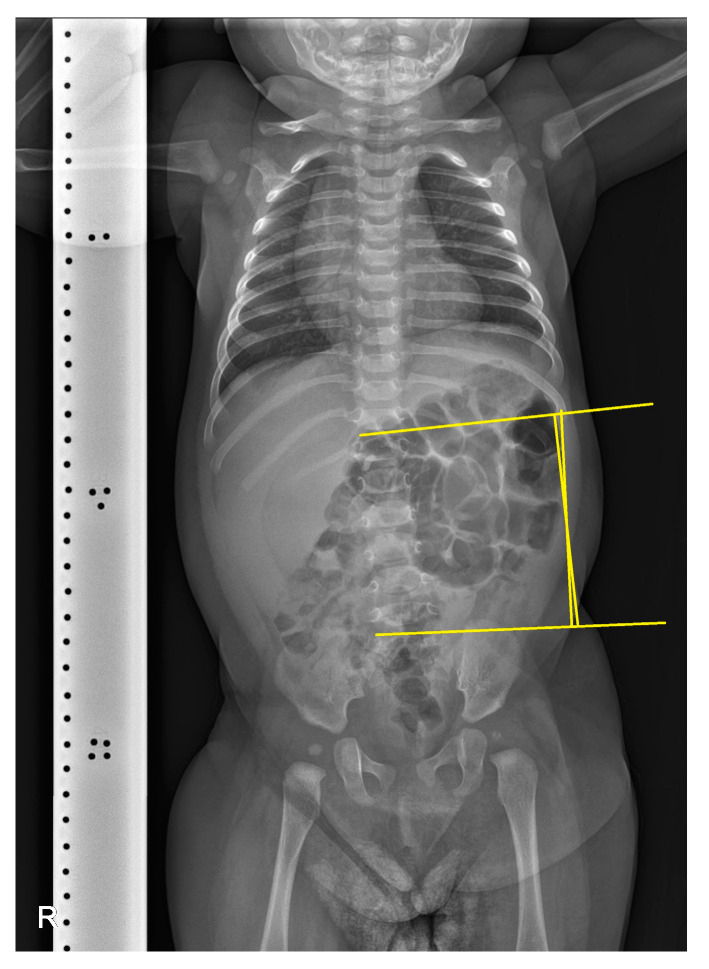
Whole-spine simple radiography. The yellow lines indicate the Cobb angle of the thoracolumbar curve, which measures 12 degrees, suggesting mild thoracolumbar scoliosis.

A simple radiographic evaluation was performed to identify any other related complications. To rule out vertebral anomalies, particularly high cervical vertebrae deformations—which could be a cause of congenital torticollis—we examined the cervical spine [9]. The cervical spine simple radiograph was obtained in the anteroposterior view and lateral-extension view (Figure 3). No bony abnormalities or airway obstructions were detected. Whole-spine simple radiography revealed mild right-side thoracolumbar scoliosis (Figure 4). The Cobb angle of the thoracolumbar curve was 12 degrees. Both scanography and pelvic simple radiography revealed no abnormalities in the lower limb and hip regions.

To rule out congenital genetic disorders and tumorous conditions, the baby was referred to the pediatrics department. No definite palpable mass was observed in the head and neck area, and the head circumference was 42.5 cm (90th–95th percentile for age). The baby exhibited no developmental delays or facial abnormalities. Abdominal ultrasonography was performed to check for other abnormalities frequently observed in genetic and tumorous conditions, such as visceromegaly and abdominal mass [2]. The most common malignant tumorous conditions associated with hemihyperplasia are Wilms tumor and hepatoblastoma; however, other tumors like neuroblastomas, adrenocortical tumors, and sarcomas can also occur [1,4]. Additionally, no bony abnormalities, which could be a possible sign of sarcoma, were found on radiography of the hip region and lower limbs. Several cystic lesions smaller than 1 cm were observed on the right ovary. Laboratory blood tests and genetics testing were refused by the parents. Due to the caregivers’ refusal of blood laboratory testing, we could not examine levels of alpha-fetoprotein and other tumor markers but no visible mass was found in the patient, so the likelihood of a tumorous condition was considered low. After 6 months of follow-up, no tumorous conditions were detected using radiographic evaluation. Nevertheless, tumorous conditions related to hemihypertrophy can develop until adolescence, so the patient was recommended to receive follow-up examinations until the age of 6 years [4].

Following the first visit, physical therapy was continued to release the soft tissue of the right neck muscle. A skilled pediatric therapist performed passive range-of-motion exercises, active range-of-motion exercises, muscle strengthening of the unaffected side, symmetrical movement-development exercises, myofascial release, and whole-body balance training [10]. Therapy was performed twice a week for 30 min each session [10]. At a 2-month follow-up visit, no remarkable change in right-side hemihyperplasia from the initial visit was observed. There were no developmental delays observed during follow-up. At 3 months after the first visit, the baby’s neck range of motion was improved, and neck ultrasonography revealed an improvement in torticollis. The thickness of the SCM muscle at the right mid-level had decreased from 12.9 mm to 10.7 mm, an 18% decrease compared to the initial evaluation. In addition, the thickness of the right SCM muscle at the clavicular and sternal insertions decreased to 5.6 and 2.3 mm, respectively, compared to the initial evaluation, where they measured 10.9 and 2.5 mm. These reductions indicate a positive response to treatment during the 3-month follow-up period. Along with the improvement in torticollis, the baby’s hemihyperplasia exhibited mild equivocal improvement. The mid-arm circumference on the right side was 159 mm, and on the left side, it was 144 mm. The mid-thigh circumference on the right side was 263 mm, and on the left side, it was 235 mm. The difference between both sides decreased to 10% compared to the 12% difference observed during the initial evaluation. The baby discontinued physical therapy and was recommended to visit every 6 months for follow-up.

## 3. Discussion

The causes of hemihypertrophy can vary from idiopathic hemihypertrophy to underlying malignant tumorous conditions. The condition can be idiopathic, and the possible underlying conditions include Proteus syndrome, hemihyperplasia–multiple lipomatosis syndrome, Klippel–Trenaunay–Weber syndrome, familial hypertrophy, chronic hyperemia, neurofibromatosis, lymphatic disorders, vascular disorders, dysmorphic syndromes such as Beckwith–Wiedemann syndrome, and Wilms tumor. Many cases of hemihypertrophy present with symptoms such as familial history, neuropathic symptoms, visible physical defects, or other radiologic findings. A thorough clinical evaluation is required to determine whether the hemihypertrophy is of primary idiopathic etiology or secondary to underlying disease. Physical screening is important to differentiate the issue from other causes. The diagnostic criteria for Proteus syndrome include the presence of connective tissue nevi, also known as cerebriform connective tissue nevi, which are frequently found on the palms and soles of affected individuals [1]. These nevi are typically characterized by thick, raised, and grooved skin that resembles the surface of the brain; therefore, they are also referred to as cerebriform connective tissue nevi. Along with other clinical and radiographic features, the presence of these nevi is essential for accurate diagnosis of Proteus syndrome. Other possible causes of hemihypertrophy can have associated features of multiple lipomas and vascular malformations. Conditions like Klippel–Trenaunay–Weber syndrome and hemihyperplasia–multiple lipomatosis syndrome are examples of conditions where hemihypertrophy is accompanied by vascular malformations and multiple lipomas [1]. Also, skin features like café au lait spots, lisch nodules, and axillary freckling or gross abnormalities such as omphalocele, large tongue, and ear creases are associated with other conditions. These skin features and gross abnormalities are important clinical symptoms of neurofibromatosis and Beckwith–Wiedemann syndrome [1]. A thorough examination and evaluation are necessary to differentiate idiopathic hemihypertrophy from hemihypertrophy with underlying conditions, allowing appropriate management. Our patient did not exhibit other visible physical findings associated with other underlying conditions. Blood laboratory and genetic test results may also be examined and used for diagnosis. Even though underlying conditions of hemihypertrophy are diagnosed using a set of gross clinical features, diagnosis of some conditions is aided by genetic tests and blood laboratory tests.

Although idiopathic muscular torticollis was prominent, it can be viewed as a secondary change due to ipsilateral hemihypertrophy. According to a previous study by Hwang et al. [11], the average thickest part of the unaffected SCM muscle was 5.9 ± 1.1 mm, no more than 20% greater than that of the unaffected side. In this patient, the thickness values of the unaffected and affected sides were 4.7 and 12.9 mm, respectively. This discrepancy suggests that the SCM muscle was affected by ipsilateral hemihypertrophy.

There were no other underlying causes identified in this case that could affect the SCM muscle; however, the thickness of the affected SCM muscle was significantly thicker than the average reported by Hwang et al. As hemihypertrophy is defined as regional body growth asymmetry caused by differences in the growth of bone, soft tissue, or both, considering the definition of ipsilateral hemihypertrophy, hemihypertrophy could be the cause of muscular torticollis in this patient. After a few months of physical therapy, the remaining torticollis exhibited improvement, as did the hemihypertrophy. Without any other treatment, we presume that the treatment of torticollis had a beneficial effect on hemihypertrophy. The improvement of both conditions may suggest a relationship between muscular torticollis and hemihypertrophy in this patient. Moreover, radiologic and ultrasonographic evaluations did not reveal any sign of tumors or other conditions.

Idiopathic congenital hemihypertrophy is a rare condition that may result from intrinsic congenital growth excess or faulty cell division of the zygote. Isolated hemihypertrophy is another term used for this condition. There are no definite criteria for diagnosis, and this condition is characterized by asymmetric regional body overgrowth without any other underlying diagnosis. In the previous study, it was mentioned that the growth discrepancy must be greater than 5% [4]. To make the diagnosis, other causes of hemihypertrophy, such as Proteus syndrome, Beckwith–Wiedemann syndrome, neurofibromatosis, and other conditions, must be differentiated and ruled out [2]. This condition typically affects soft tissues and musculoskeletal structures, while bone age and development are usually normal. Bone growth is expected to be consistent with age on both the affected and unaffected sides; therefore, the unaffected side does not always “catch up” in growth during development [12]. Idiopathic hemihypertrophy is typically most evident from birth, and there can be variations in growth patterns among patients. In some cases, the difference between the affected side and the unaffected side may be subtle. As in this case, hemihypertrophy can be present as torticollis. The condition can lead to progressive disproportion or “catch-up” growth of the unaffected side. However, true idiopathic hemihypertrophy typically involves growth without a change in the asymmetry of body proportions [12]. Idiopathic hemihypertrophy is a relatively benign condition but could lead to many social and psychological issues resulting from unsightly cosmetic conditions. The leg-length discrepancy is a common adulthood problem related to idiopathic hemihypertrophy. Symptom-related treatment is required for appropriate growth. Although there are typically no other symptoms, regular follow-up examinations for intra-abdominal masses are mandatory. As malignant conditions associated with hemihypertrophy can develop until adolescence, ultrasound examinations should be performed every 3–6 months.

## 4. Conclusions

Congenital hemihyperplasia in an infant is rare, without many reported cases; moreover, regional body growth asymmetry varies among patients, making it difficult to recognize and diagnose hemihyperplasia. Hemihyperplasia could be associated with several genetic disorders and tumorous conditions. Underlying diseases can lead to the development of malignant conditions. Early surveillance until the age of adolescence is essential; however, torticollis is a relatively common condition in infants and is easily recognized by caregivers and clinicians. When torticollis is observed as a symptom of hemihypertrophy, it can aid in the early detection of hemihypertrophy and possible underlying conditions. We report an infant with hemihyperplasia and muscular torticollis on the ipsilateral side. This case indicates that clinicians should consider muscular torticollis as a symptom of hemihyperplasia, and patients with hemihyperplasia have an increased risk of embryonal tumorous conditions and genetic disorders. To detect new-onset tumors related to hemihypertrophy, follow-up evaluations should be performed every 3–6 months. Regular monitoring is crucial to identify any potential changes in the condition, especially any related to tumor growth. Clinicians should not overlook the possibility of hemihyperplasia and underlying conditions when torticollis is detected.

## Data Availability

Not applicable.
